# The utility of non‐axial treatment beam orientations for lower lobe lung cancers

**DOI:** 10.1120/jacmp.v11i1.3010

**Published:** 2010-01-28

**Authors:** Brian P. Quaranta, Shiva K. Das, Timothy D. Shafman, Kim L. Light, Lawrence B. Marks

**Affiliations:** ^1^ 21st Century Oncology Asheville NC; ^2^ Department of Radiation Oncology Duke University Medical Center Durham NC; ^3^ 21st Century Oncology Providence RI; ^4^ Department of Radiation Oncology University of North Carolina at Chapel Hill Chapel Hill NC USA

**Keywords:** lung cancer, 3D planning, non‐axial beams, radiation therapy

## Abstract

Traditional treatment beams for non‐small–cell lung cancer are limited to the axial plane. For many tumor geometries, non‐axial orientations appear to reduce the dose to normal tissues (e.g. heart, liver). We hypothesize that non‐axial beams provide a significant reduction in incidental irradiation of the heart and liver, while maintaining adequate target coverage. CT scans of twenty‐four patients with lower lobe lung cancers were studied. For each patient, an opposed oblique axial beam pair and a competing non‐axial opposed oblique pair were generated, both off‐cord. The competing plans delivered comparable doses/margins to the GTV. DVHs and integral doses were computed for all structures of interest for the two competing plans. The integral dose was compared for axial and non‐axial beams for each contoured organ using a paired t‐test. Dose to the heart was significantly lower for the non‐axial plans (p=.0001). For 20/24 patients, the integral heart dose was reduced by using non‐axial beams. In those patients with tumors located in the inferior right lower lobe, a lower dose to the liver was achieved when non‐axial beams were used. There were no meaningful differences in dose to the GTV, lungs, or skin between axial and non‐axial beams. Non‐axial beams can reduce the dose to the heart and liver in patients with lower lobe lung cancers. Non‐axial beams may be clinically beneficial in these patients and should be considered as an option during planning.

PACS number: 87.55.de

## I. INTRODUCTION

A fundamental goal of radiation therapy is to increase the therapeutic ratio by delivering adequate tumor dose while simultaneously minimizing dose to adjacent normal tissues. This is particularly difficult to achieve in patients with lung cancer, since the tumor is often located adjacent to vital organs such as the heart, spinal cord, esophagus, liver, and healthy lung. Conventional treatment planning for non‐small–cell lung cancer (NSCLC) includes initial anteroposterior and posteroanterior (AP‐PA) fields treated to the dose accepted for spinal cord tolerance, followed by oblique boost fields designed to avoid the spinal cord. These fields have traditionally been set up in the axial plane. There has been little change in this approach for decades.

**Figure 1:**
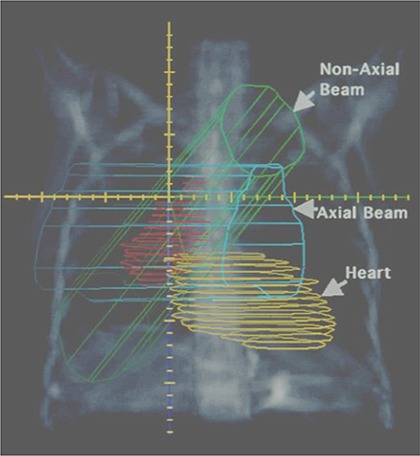
Potentially reducing the field size through rotation of the beam out of the axial plane. This approach may better align the central ray with the long axis of the GTV. This image also demonstrates the exclusion of the heart with the non‐axial beam.

Examination of the three‐dimensional relationship between the lung tumor and adjacent vital organs, particularly the heart and liver, indicates that it is often possible to better exclude these adjacent structures from the field if the beam is rotated out of the axial plane (Fig. 1). This may be expected to increase the distance within the body through which the radiation beam would have to travel. The assumption therefore would be that the reduced dose to the heart and/or liver would be accompanied by increased dose to normal lung tissue. However, the three‐dimensional shape of these tumors is often such that rotating the beam out of the axial plane may better align the beam with the long axis of the gross tumor volume (GTV), thereby reducing the field size. This is especially true in the case of lower lobe tumors with involvement of the mediastinal lymph nodes, where the beam can be aligned such that the involved lymph nodes fall into the “shadow” of the primary tumor.

We therefore hypothesize that for unresectable non‐small–cell lung cancer of the lower lobes, rotation of the boost field out of the axial plane will: a) decrease the dose to critical normal structures such as the heart and liver, b) slightly increase the beam path length through the patient, and c) not demonstrably increase dose to the lung since there might be a reduction in field size to offset the increased path length.

## II. MATERIALS AND METHODS

The CT images of 24 consecutive patients with unresectable lower lobe lung cancers that had 3D radiation treatment planning at Duke were studied. All patients underwent CT scanning in a customized immobilization cradle in the treatment position, with images taken at 0.5 cm intervals from (approximately) the skull base through the iliac crest. Each patient's CT images were incorporated into the PLUNC (Plan – University of North Carolina) 3D planning system and used to define the gross target volume (GTV), heart, lungs, spinal cord, esophagus, and skin. All patients were given esophageal contrast at the time of CT scanning to aid in defining the esophagus. The outside border of the esophagus was then used to define the contour. Recognizing the intimate association of the heart with the great vessels, our convention was to define “heart” as the cardiac chambers and great vessels located inferiorly to the pulmonary artery. In those patients with tumor near the diaphragm, the entire extent of their liver was scanned and this was identified.

Patients are usually treated initially with an AP‐PA field to approximately 44–46 Gy, followed by off‐cord boost fields to take the final dose to 66 Gy. Competing off‐cord boost fields are the subject of the comparisons in this report. For each patient, an opposed oblique axial beam pair was defined to include the entire GTV with a 1.5 cm margin, while simultaneously avoiding the spinal cord with a 0.5 cm margin (cord avoidance was given priority when the two goals were in conflict). An automatic block design tool was used to achieve the above listed margins. The fields were designed to minimize involvement of the heart and liver to the greatest extent possible.

A competing non‐axial opposed oblique pair was then defined in an attempt to reduce dose to adjacent normal tissues while maintaining the same margins outlined above. The extent of non‐axial deviation was determined based upon visual inspection of the beam on the CT data set, and was limited by the physical constraints of the treatment machine (i.e. the angles were chosen so that the gantry did not collide with the patient or table). A 3D dose calculation reflecting tissue density inhomogeneity correction was performed for each plan. Dose was defined as 100% at the isocenter. Customized conformal blocks as drawn in the planning system were used during the calculations. For each patient, the minimum and mean doses to the GTV for the axial and non‐axial plans were verified to be similar to assure that the comparison was reasonably valid.

The primary endpoints for the study were dosimetric measures from structures of interest. Dose‐volume histograms (DVHs) were computed for all structures of interest for the two competing boost plans (axial and non‐axial). From each DVH, a variety of dosimetric metrics for the structures of interest were used to compare the two treatment plans. The percent of normal tissue receiving 20%, 50%, 80%, and 100% of the isocenter dose were compared for heart, lung, liver, esophagus, and skin. Percent of the spinal cord receiving 5% and 10% was measured (no portion of the spinal cord received > 20% of the dose). For the GTV, the minimum and maximum doses delivered, and the percent of the GTV getting 100% of the prescribed dose, were recorded. The integral dose was then calculated for each organ from its differential dose volume histogram as the summation of the product of the midpoint dose of each dose bin and the volume corresponding to that bin (dose is normalized to 100% at the isocenter, and volume is in cc units). The above parameters were compared for axial and non‐axial beam boost plans using the paired t‐test.

**Figure 5:**
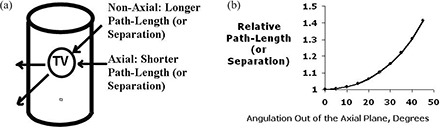
Schematic illustration of the longer path length, or greater separation, for non‐axial vs. axial beams (a); graph (b) demonstrates that the increase in path length (calculated using the cosine of the angle created by the deviation of the selected path from the axial plane) should be minimal when the off‐axis deviation is less than approximately 30°. Clinically, this path length is commonly referred to as the patient's “separation.”

In addition, the characteristics of the axial and non‐axial beams were compared, including separation and unblocked field size. The separation is the “thickness” of the patient along the central ray of the treatment beam – also referred to as the “path‐length” of the beam in the patient (see discussion, Fig. 5). The unblocked field size does not consider the effect of customized blocks, and thus does not accurately reflect the irradiated volume. However, it provides an estimate of the maximum dimensions of the GTV, as seen in the BEV.

## III. RESULTS

### A. Patient characteristics

The patients’ tumors were staged as follows: Tx=1, T1=4, T2=11, T3=3, T4=5. N stage: Nx=2, N0=5, N1=0, N2=12, N3=5. The average tumor volume (T+N) was 145 cc (range 18–542).

### B. Plan comparison: dosimetric parameters

Details of the dosimetric comparisons are presented in Table 1. There were no significant differences, for any of the structures, between the two types of plans at any of the specific volumes for which percent dose delivered was compared. However, the percent of the heart receiving different threshold doses generally appeared lower for the non‐axial beams, and the percent of lung generally appeared minimally higher.

**Table 1:** Dosimetric comparison of axial vs. non‐axial beams.

	*Axial Beams*	*Non‐Axial Beams*	*p value*
% Heart receiving 20% of the dose	37.3	22.0	NS
% Heart receiving 50% of the dose	28.4	16.0	NS
% Heart receiving 80% of the dose	23.8	12.3	NS
% Heart receiving 100% of the dose	13.4	6.1	NS
% Lung receiving 20% of the dose	26.8	28.4	NS
% Lung receiving 50% of the dose	21.6	23.3	NS
% Lung receiving 80% of the dose	17.6	19.7	NS
% Lung receiving 100% of the dose	11.6	12.9	NS
% Esophagus receiving 20% of the dose	40.6	42.3	NS
% Esophagus receiving 50% of the dose	34.8	35.8	NS
% Esophagus receiving 80% of the dose	29.8	30.4	NS
% Esophagus receiving 100% of the dose	12.2	14	NS
% Spinal cord receiving 5% of the dose	3.0	4.5	NS
% Spinal cord receiving 10% of the dose	0.6	0.8	NS
% Spinal cord receiving 20% of the dose	0	0	NS

The comparisons of integral dose are summarized in Table 2. Integral dose to the heart was significantly lower for the non‐axial plans (p=0.0001). Twenty out of 24 non‐axial plans achieved at least some reduction in heart dose. This reduction was > 67% in nine cases.

**Table 2:** Comparisons of integral dose.

*Mean Integral Dose to Organ* ^a^	*Axial Beam*	*Non‐axial Beam*	*No. of Pts with Lower Dose in Non‐axial Fields*	*p‐value*
GTV	14,698	14,691	13/24	0.9
Heart	19,077	10,725	20/24	0.0001
Lungs	88,598	91,027	11/24	0.5
Esophagus	1,161	1,188	7/24	0.6
Spinal cord	46.5	53.4	4/24	0.003
Liver	3,1021	10,525	4/4	0.1
Skin	11,052	10,895	10/24	0.6

^a^ Units for mean integral dose are “%dose x cc”.

**Figure 2:**
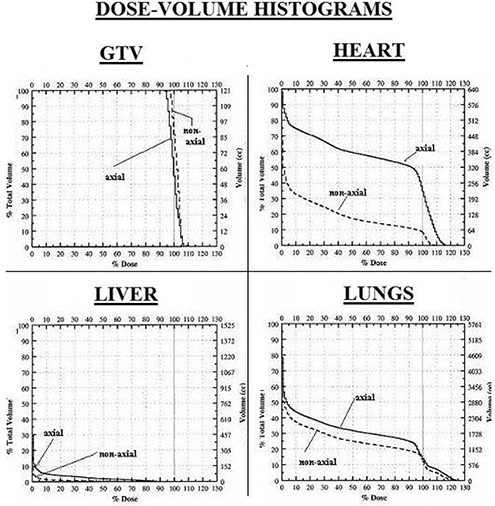
Sample dose‐volume histograms for the GTV, heart, normal lung, and liver for one of the studied patients.

There was a statistically significant increase in mean integral dose to the spinal cord using non‐axial beams. However, this difference is unlikely to be clinically relevant since the doses involved were small; maximum cord dose was 8.2% vs. 10.9% (p=0.04) for axial and non‐axial beams, respectively, with corresponding mean cord doses of 1.3% and 1.5% (p=0.005). There was no significant difference in integral dose to the liver between plans (p=0.1). This is likely attributable to the small number of patients whose livers had been completely scanned (n=4). All four of these patients achieved a lower dose to the liver when non‐axial beams were used. The dose reduction using axial plans was 65%, 38%, 33%, and 22% in these cases. Figure 2 shows dose‐volume histograms for the GTV, heart, lungs and liver for a sample patient.

**Figure 3:**
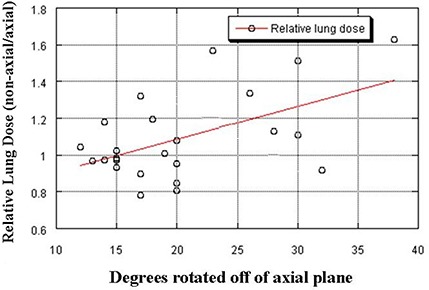
The relationship between relative normal lung dose (integral lung dose using non‐axial plan/integral lung dose using axial plan) and degree of rotation off of the axial plane is shown. A trend toward increasing dose to the normal lung tissue with increasing rotation off the axial plane is seen ($R^{2}=0.28$, p=0.008).

Figure 3 demonstrates the relationship between integral dose to the normal lung vs. degree of rotation off the axial plane, indicating a trend toward increasing relative lung dose (non‐axial/axial) with increasing off‐axis rotation. Despite this trend, the average values were not significantly different between the two plans in this study.

### C. Plan comparison: beam characteristics

The non‐axial beams were rotated 17.5°−32° out of the axial plane (mean 18.5, SD 4.86). There were five patients for whom rotation of the beam out of the axial plane was unable to achieve any dosimetric benefit on any of the tested structures, and this was obvious at the time of planning. Among all tested patients, there was no difference in mean field size between the two sets of beams, which measured $122\;{\text{cm}}^{2}$ for both axial and non‐axial beams.

Similarly, there was no difference in separation (mean 29.5 cm and 29.4 cm for axial and non‐axial plans, respectively).

## IV. DISCUSSION & CONCLUSIONS

The results of this study indicate that rotation of the treatment beams out of the axial plane may decrease the dose delivered to the heart and liver without compromising the dose delivered to the GTV, lungs, esophagus, and spinal cord in a clinically relevant manner.

Numerous studies have demonstrated that patients undergoing radiation therapy for breast cancer suffer an increased rate of heart disease, and that the incidence of this complication is related to dose. Gyenes et al.^(1)^ evaluated late cardiac effects in patients with breast cancer randomized to pre‐ or postoperative radiation vs. surgery alone. They found that cardiac mortality was positively correlated with the cardiac dose‐volume; patients in the high dose‐volume group exhibited a hazard ratio for cardiac death of 2.0 (95% CI 1.0‐3.9, p=0.04) relative to that of the surgical controls. Stewart et al.^(2)^ reviewed the literature for late heart toxicity as a result of radiation therapy (based primarily on patients breast cancer and Hodgkin's disease) and concluded that “it is clear that there is a dose response for pericardial and myocardial disease.” Several other authors have concluded that mediastinal irradiation for treatment of Hodgkin's disease resulted in increased cardiac disease^(3)^ and mortality.^(^ ^4^ ^,^ ^5^ ^,^ ^6^ ^)^

These data from patients with breast cancer and Hodgkin's disease may be extrapolated to patients with lung cancer. In practice, this may not be clinically relevant for most patient groups, since cancer‐related deaths severely limit survival times. In the postoperative setting, however, where survival rates for lung cancer are relatively good, the cardiotoxic effects of RT may be more readily apparent. In fact, in a randomized trial assessing the usefulness of postoperative RT for non‐small–lung cancer, Dautzenberg et al.^(7)^ demonstrated a 3:1 ratio of intercurrent cardiac deaths in the irradiated group vs. unirradiated controls. Currently, short overall survival time limits our ability to assess late effects of irradiation upon the heart in patients with NSCLC. However, if newer therapies for lung cancer improve outcomes, the long‐term cardiac effects of treatment may become more evident.

Scant literature specifically addresses the use of non‐axial (or non‐coplanar) beams in lung cancer. Wustbauer et al.^(8)^ and Pirzkall et al.^(9)^ describe attempts to use alternate beam arrangements to treat NSCLC to high doses while avoiding normal tissues; however, both of these studies involve multiple beams in the axial plane. Derycke et al.^(10)^ attempted to improve the therapeutic ratio by using non‐coplanar beams in a 1997 study. They compared traditional 2D planning, using AP‐PA large field beams to 46 Gy followed by an off‐cord oblique boost, vs. 3D planning designed to deliver the entire treatment via three or four non‐coplanar beams. The 3D technique allowed delivery of significantly higher doses to the GTV while staying within lung and spinal cord tolerance. They applied a 3D intensity modulation class solution (3D‐BIM) to the six cases in which normal tissue tolerance had limited the maximum GTV dose to 80 Gy or less. Use of the 3D‐Beam Intensity Modulation (BIM) technique enabled the planners to deliver higher doses to the GTV in all six cases without exceeding lung or spinal cord tolerance. They did not assess the doses delivered to the heart, liver, and esophagus.

**Figure 4:**
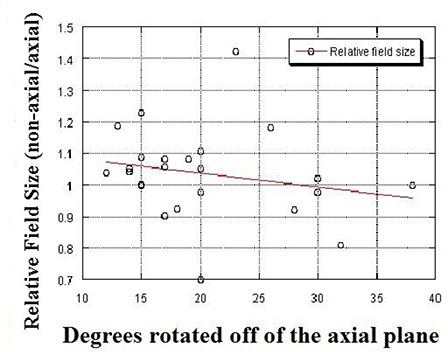
The relationship between relative field size (size of the non‐axial field/size of the axial field) vs. degree of rotation off of the axial plane is shown. There is a slight trend, albeit nonsignificant, toward decreasing size of the non‐axial field as the beam is rotated farther away from the axis ($R^{2}=0.04$, p=0.327).

Rotating the beam out of the axial plane is intended primarily to avoid adjacent normal tissues. However, a secondary intention is to reduce the field size. The GTV of patients with lower lobe tumors and associated mediastinal/hilar lymph nodes is often approximately cylindrical, with the long axis of the cylinder out of the axial plane. Thus, rotation away from the axial plane could decrease the field size needed to encompass the lesion (Fig. 4). Overall, there was no difference in the mean unblocked field size between the axial and non‐axial plans in this study. However, there was a slight trend within the non‐axial groups towards decreasing relative (non‐axial/axial) field size with increasing rotation away from the axial plane (Fig. 4). The lack of a significant difference in field size may, therefore, be a result of the generally modest degree of off‐axis rotation (mean 18.5 degrees).

Given the cylindrical shape of the chest and lungs, we were concerned that rotating the beam out of the axial plane would increase the path length through the patient and thus increase the dose to the normal lung tissue. Our study did not confirm a significant difference in path length between the axial and non‐axial beams. In retrospect, this is not surprising given geometrical considerations. Assuming a cylindrical external contour, the relative path length for the non‐axial vs. axial beam is 1/cosine X, where X is the angle formed between the non‐axial beam and the axial beam. As demonstrated in Fig. 5, this ratio approximates one for all angles less than approximately 30°.

Our data do support the suggestion that beams with a greater degree of angulation may modestly increase the lung dose (Figs 3, 5). This is a result of potential increased path length and must be kept in mind when considering significantly angled non‐axial beams.

The angle of rotation out of the axial beam is limited by technical considerations (i.e. the physical constraints of the machine). Depending on the equipment used, the desirable angle may be unobtainable due to a collision of the gantry and table or patient. In this study, we limited the beams used to those that were technically possible on our equipment; the range varied based on the degree of table rotation required to achieve the desired angle.

Non‐axial beams may also be more difficult to set up than standard axial beams. In general, increased complexity of treatment may be expected to provide more opportunities for human error. Therapists might be less familiar with using non‐axial beams, and therefore longer treatment times and an increased incidence of setup errors may occur. Similarly, physicians are accustomed to evaluating the geometric relationships of the thoracic organs when seen in the axial plane; rotating the treated ports off this axis will therefore make the portal images less familiar and more difficult to evaluate. The extent to which these difficulties will manifest themselves is difficult to quantify and obviously will vary from institution to institution, depending upon the volume of cases treated using these techniques. Computer controlled machines, patient verification systems, and improved portal imaging will mitigate these issues.

There were five patients in this study for whom rotation of the beam out of the axial plane was unable to achieve any dosimetric benefit on any of the tested structures, and this was obvious at the time of planning. However, in many cases of NSCLC arising in the lower lobes, use of non‐axial beams will be able to improve the therapeutic ratio. The treating radiation oncologist must use his or her clinical judgment to balance the potential benefits of non‐axial beams against the possible increased technical difficulty of treatment depending upon the circumstances of each case. Given the added effort associated with non‐axial beams, a quantitative comparison of axial and non‐axial beams should be considered for each patient before implementing non‐axial treatments.

## ACKNOWLEDGEMENTS

This work was supported by a grant from the Armstrong Family Foundation. Thanks to the University of North Carolina at Chapel Hill for use of the PLUNC (Plan‐UNC) treatment planning software.
